# Lack of cardioprotection by single-dose magnesium prophylaxis on isoprenaline-induced myocardial infarction in adult Wistar rats

**DOI:** 10.5830/CVJA-2015-055

**Published:** 2015

**Authors:** Christie Garson, Roisin Kelly-Laubscher, Asfree Gwanyanya, Dee Blackhurst

**Affiliations:** Department of Human Biology, Faculty of Health Sciences, University of Cape Town, Cape Town, South Africa; Department of Human Biology, Faculty of Health Sciences, University of Cape Town, Cape Town, South Africa; Department of Human Biology, Faculty of Health Sciences, University of Cape Town, Cape Town, South Africa; Department of Clinical Laboratory Sciences, Faculty of Health Sciences, University of Cape Town, Cape Town, South Africa

**Keywords:** cardiac, isoprenaline, magnesium, myocardial infarction

## Abstract

**Aim:**

Magnesium (Mg^2+^) is effective in treating cardiovascular disorders such as arrhythmias and pre-eclampsia, but its role during myocardial infarction (MI) remains uncertain. In this study, we investigated the effects of Mg^2+^ pre-treatment on isoprenaline (ISO)-induced MI *in vivo*.

**Methods:**

Rats divided into four groups were each pre-treated with either MgSO_4_ (270 mg/kg intraperitoneally) or an equivalent volume of physiological saline, prior to the ISO (67 mg/kg subcutaneously) or saline treatments. One day post-treatment, the electrocardiogram and left ventricular blood pressures were recorded. Infarcts were determined using 2,3,5-triphenyltetrazolium chloride staining, and serum markers of lipid peroxidation were measured with spectrophotometric assays.

**Results:**

Mg^2+^ pre-treatment neither altered the ISO-induced infarct size compared with ISO treatment alone (^p^ > 0.05), nor reversed the low-voltage electrocardiogram or the prominent Q waves induced by ISO, despite a trend to decreased Q waves. Similarly, Mg^2+^ did not prevent the ISO-induced decrease in peak left ventricular blood pressure or the decrease in minimal rate of pressure change. Mg^2+^ did not reverse the ISO-induced gain in heart weight or loss of body weight. Neither ISO nor Mg^2+^ altered the concentrations of lipid peroxidation markers 24 hours post MI induction.

**Conclusion:**

Although Mg^2+^ had no detrimental effects on electrical or haemodynamic activity in ISO-induced MI, the lack of infarct prevention may detract from its utility in MI therapy.

## aim

Magnesium (Mg_2+_) is used in the treatment of life-threatening cardiovascular disorders such as arrhythmias and pregnancyinduced hypertension.^1^ However, there is uncertainty regarding its role in myocardial infarction (MI), a common and lethal complication of many cardiovascular disorders.

A meta-analysis of early clinical trials showed that intravenous Mg_2+_ reduced mortality and arrhythmias in acute MI.^2^ In large trials, beneficial effects of Mg2+ were also found in the second Leicester Intravenous Magnesium Intervention trial (LIMIT-2), in which Mg_2+_ infusion preceded thrombolytic therapy,^3^ as well as in studies involving high-risk patients unfit for thrombolysis.^4^ By contrast, Mg_2+_ did not improve survival in the fourth International Study of Infarct Survival (ISIS-4) trial, in which Mg_2+_ was given after thrombolytic therapy,^5^ and in the more recent Magnesium in Coronaries (MAGIC) trial,^6^ which included high-risk patients not eligible for reperfusion therapy.

In animal studies, Mg_2+_ reduced infarct size^7^-^11^ and inhibited myocardial apoptosis^12^ under certain conditions, but not others.^13^,^14^ There is therefore a need for further experimental and clinical studies on Mg_2+_ therapy.

Mg_2+_ is proposed to modulate MI through its antithrombotic,^15^ antioxidant^16^ and anti-arrhythmic effects.^17^ Through its ability to block Ca_2+_ channels,^18^ Mg_2+_ prevents cytosolic Ca_2+_ overload,^19^ and decreases both systemic and coronary vascular tone.^20^ At a cellular level, Mg_2+_ is an essential co-factor for several enzymes, including those involved in ATP synthesis and utilisation. Furthermore, it is a co-factor for ATP activity in the form of MgATP.^21^ Mg_2+_ preconditions the myocardium through the activation of ATP-dependent K^+^ channels^22^ and also confers resistance to mitochondrial membrane depolarisation,^23^ thereby minimising mitochondrial Ca_2+_ overload.

Mitochondrial Ca^2+^ overload attenuates ATP synthesis and augments ATP hydrolysis, particularly that of MgATP.^24^ In the form of an orotate salt, Mg^2+^ prevents the opening of the mitochondrial permeability transition pore, which is lethal to cells.^25^ However, while acute MI is associated with decreased serum Mg^2+^ levels,^26^ the conditions under which Mg^2+^ is cardioprotective remain uncertain.

The synthetic catecholamine, isoprenaline (ISO), has been widely used to induce infarcts mimicking human global MI. Overstimulation of β-adrenergic receptors by ISO induces MI through the generation of free radicals,^27^,^28^ intracellular Ca^2+^ overload,^29^ and apoptosis.^30^ In addition, the stress due to the MI itself causes further release of catecholamines and worsens the MI. Catecholamine-mediated β-adrenergic receptor stimulation also induces Mg^2+^ efflux,^31^,^32^ thereby potentially depleting intracellular Mg^2+^. Mg^2+^ is also known to inhibit catecholamine release.^33^

The framework of the current study was therefore to investigate the role of Mg^2+^ prophylaxis in cardiac stress conditions, in which extracellular and intracellular Mg^2+^ homeostasis may be altered. We investigated the effects of Mg^2+^ pre-treatment on cardiac morphological, electrical and haemodynamic changes, and on the lipid peroxidation profile in a rat model of acute MI induced by ISO.

## Methods

Adult male Wistar rats, weighing 250–300 g, were obtained from the University of Cape Town animal unit and housed in an air-conditioned animal facility under standard laboratory conditions (12-hour light/dark cycle, illumination of 323 lux and temperature of ~22°C). The rats were fed standard rat chow (Afresh Vention 1, Cape Town, South Africa) and had free access to food and water.

Experimental procedures were approved by the animal ethics committee of the Faculty of Health Sciences, University of Cape Town. All protocols were carried out in compliance with the *Guide for the Care and Use of Laboratory Animals* [NIH Publication No. 85 (23), revised 1996].

## Animal procedures and experimental protocol

**Fig. 1.**
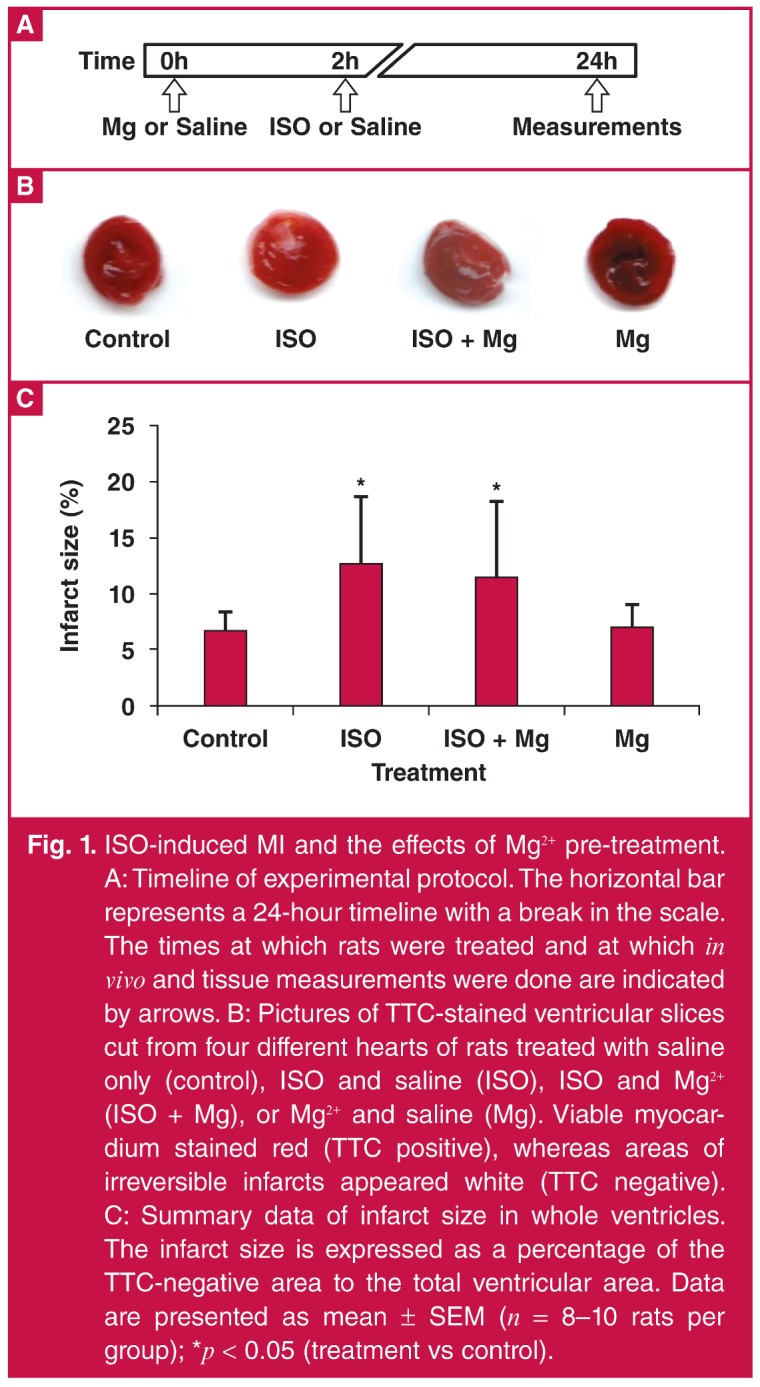
ISO-induced MI and the effects of Mg^2+^ pre-treatment. A: Timeline of experimental protocol. The horizontal bar represents a 24-hour timeline with a break in the scale. The times at which rats were treated and at which *in vivo* and tissue measurements were done are indicated by arrows. B: Pictures of TTC-stained ventricular slices cut from four different hearts of rats treated with saline only (control), ISO and saline (ISO), ISO and Mg^2+^ (ISO + Mg), or Mg^2+^ and saline (Mg). Viable myocardium stained red (TTC positive), whereas areas of irreversible infarcts appeared white (TTC negative). C: Summary data of infarct size in whole ventricles. The infarct size is expressed as a percentage of the TTC‑negative area to the total ventricular area. Data are presented as mean ± SEM (*n* = 8–10 rats per group); *p < 0.05 (treatment vs control).

Thirty-five rats were divided into four groups and treated according to the experimental protocol described below, for which the timeline is shown in (Fig. 1A). Subcutaneous (sc) injection of ISO at 67 mg/kg in rats is known to produce histologically detectable MI within 24 hours.^34^ In preliminary tests, we observed that using higher doses of ISO, such as 85 mg/kg and above, resulted in high mortality rates in our rats.

The ISO-induced MI group (*n* = 9) was pre-treated with intraperitoneal (ip) injection of physiological saline (2.7 ml/kg) two hours prior to injection with ISO (67 mg/kg sc), and the ISO + Mg^2+^ group (*n* = 10) was pre-treated with MgSO_4_ (270 mg/kg ip), which is effective in neuroprotection,^35^ two hours prior to injection with ISO (67 mg/kg sc). The two-hour wait was meant to avoid possible direct interactions between Mg^2+^ and ISO when co-administered at Mg^2+^ peak levels. It was also to allow adequate time for the onset of any downstream cellular effects of Mg^2+^ treatments that may have occurred prior to the induction of MI, but before the return of serum Mg^2+^ to the baseline levels expected after 3.5 hours.^35^ The Mg^2+^ group (*n* = 8) was pre-treated with MgSO_4_ (270 mg/kg ip) two hours prior to saline injection (3.3 ml/kg sc), and the control group (*n* = 8) was injected with two drug-equivalent volumes of saline (ip and sc) two hours apart.

Haemodynamic and other *in vivo*. measurements were performed under anaesthesia 24 hours after the treatments. Rats were anaesthetised with sodium pentobarbitone (60 mg/kg ip), intubated and mechanically ventilated with room air at 70 strokes/min and 2.5 ml/stroke using a rodent ventilator (Model 681, Harvard Apparatus, Holliston, Massachusetts, USA). The depth of anaesthesia was adjusted to achieve loss of pedal withdrawal reflexes, and top-up doses of sodium pentobarbitone (12 mg/kg ip) were administered where necessary. Rats were placed on a heating pad (37°C) and the body temperature was monitored using a rectal probe connected to a T-type pod transducer (ML312, ADInstruments, Bella Vista, Australia).

## Electrocardiogram and haemodynamic recordings

Lead II of a three-lead surface electrocardiogram (ECG) was used to monitor cardiac electrical changes and compute heart rate, and was recorded via an animal bio-amplifier (ML136, ADInstruments, Bella Vista, Australia). Left ventricular blood pressure was measured with a Millar Mikrotip manometer (SPC320, Millar, Houston, Texas, USA) inserted through the right carotid artery in the neck and connected to a bridge amplifier (ML221, ADInstruments, Bella Vista, Australia). To prevent drift of pressure from the baseline during recording, the manometer was cleaned with a physiological detergent (Terg-A-Zyme, Alconox, New York, USA) and zeroed in water at 37°C. Clot formation around the manometer was prevented by injecting the rats with heparin (100 IU) intravenously.

After 20 minutes of recordings, the heart was rapidly excised and retrogradely flushed with cold (4°C) saline through a cannula inserted into the aorta. The heart was then blotted, weighed and stored at –20°C for histochemical staining. To prevent damage of the epicardium due to freeze-drying, the hearts were wrapped in cling film before being frozen. Pulmonary trunk blood was collected during heart excision and centrifuged to obtain plasma, which was snap frozen in liquid nitrogen and stored at –80°C for lipid peroxidation studies. The other organs such as the liver, lungs, kidneys and adrenal glands were also excised and weighed.

Haemodynamic parameters, ECG and temperature measurements were recorded onto the computer using the PowerLab 4/30 data-acquisition system and the LabChart 7.3.5 software (ADInstruments, Bella Vista, Australia). Haemodynamic and ECG data were analysed using LabChart 7 Pro BP and ECG analysis modules (ADInstruments, Bella Vista, Australia). The ECG analysis module was preset to the rat waveform and Bazett’s formula (QTc = QT/√RR) was used to calculate the QT interval, corrected for heart rate (QTc).

## Infarct size quantification

A series of 2-mm-thick ventricular transverse slices of the frozen heart were cut from apex to base and thawed for 2,3,5-triphenyltetrazolium chloride (TTC) staining. The slices were incubated in a solution of 1% TTC in phosphate buffer (pH 7.4) at 37°C for 20 minutes and agitated periodically while protected from light. The slices were then washed with the buffer and fixed with 10% formalin to enhance contrast and stored in the dark at room temperature for 24 hours.

The slices were placed between two glass slides and scanned on both sides using a flatbed scanner. The ventricular infarct size was measured as an average of the TTC-negative areas on the slices from each heart using ImageJ software (Version 1.44p, NIH, USA) and was expressed relative to the total ventricular area.

## Lipid peroxidation assays

Markers of oxidative stress, measured as by-products of lipid peroxidation, namely conjugated dienes (CD) and thiobarbituric acid-reactive substances (TBARS), were quantified in the plasma using spectrophotometric assays. CD assays were carried out using the methods described by Esterbauer.^36^

Briefly, 100 µl of plasma was added to 405 µl chloroform: methanol (2:1). After centrifugation at 6 000 g for 15 minutes, the top aqueous layer was removed and the organic layer was isolated and dried under nitrogen. Cyclohexane (250 µl) was added to solubise the dry organic residue and the absorbance was read at 234 nm on a spectrophotometer (Spectramax Plus 384, Molecular Devices and Labotec, Johannesburg, South Africa) using Softmax Pro (Version 4.4) software. A molar extinction coefficient of 2.95 × 10^4^ /M/cm was used.

TBARS were measured using the method described by Jentzsch *et al*.^37^ Briefly, 6.25 µl of 4 mM butylated hydroxytoluene/ethanol and 50 µl of 0.2 M ortho-phosphoric acid were added to 50 µl of plasma samples and vortexed. TBA reagent (6.25 µl), dissolved in 0.1 M NaOH, was added and the mixture was centrifuged at 3 000 g for two minutes to collect small volumes at the bottom of the Eppendorf tube. The volumes were heated at 90°C for 45 minutes, placed on ice for two minutes and then left at room temperature for five minutes before *n*-butanol (500 µl) was added. Phase separation was enhanced by the addition of 50 µl of saturated NaCl.

The samples were vortexed and centrifuged at 12 000 *g* for two minutes and 300 µl of the top butanol phase was transferred into wells and read at 532 nm on the spectrophotometer. A molar extinction coefficient of 1.54 × 10^5^ /M/cm was used. The measurements of CD and TBARS were performed in triplicate and the mean value was taken as the final result.

## Chemicals and reagents

ISO and MgSO_4_ were each dissolved in physiological saline. The TTC buffer was made up of one part 0.1 M monosodium phosphate (NaH_2_PO_4_) and four parts 0.1 M disodium phosphate (Na_2_HPO_2_). Sodium pentobarbitone was purchased from Kyron Laboratories, Johannesburg, South Africa. All other drugs and chemicals were obtained from Sigma, Johannesburg, South Africa.

## Statistical analysis

Data are expressed as mean ± standard error of the mean (SEM), with *n* indicating the number of rats studied under each condition. Statistical analysis was conducted using Prism 5 (GraphPad, USA). A box-plot analysis was conducted to exclude outliers. The distribution of data was checked using the Kolmogorov–Smirnov, D’Agostino and Pearson, and the Shapiro–Wilk normality tests. Differences among multiple groups were evaluated using analysis of variance (ANOVA), followed by a Tukey *post hoc* test. For data not normally distributed and for normally distributed data that failed the Bartlett’s test, a Kruskal–Wallis test was conducted followed by a Dunns *post hoc* test; *p* ≤ 0.05 was taken as the threshold for statistical significance.

## Results

## Effects of Mg_2+_ on ISO-induced infarct size

(Fig. 1B) shows typical pictures of TTC-stained ventricular slices cut from four different hearts. Whitish-looking, TTC-negative areas were more prominent in the ISO-treated hearts, indicating the presence of irreversible infarction. The infarcted areas were patchy and more diffusely located on the myocardium, consistent with a global type of infarction compared to well-demarcated infarcts due to coronary artery ligation.

In contrast to the effects of ISO, the control and Mg^2+^-only treated hearts appeared mostly red (TTC positive), suggesting tissue viability. The quantification of infarct size in whole ventricles is summarised in Fig. 1C, which confirms that ISO induced significant increases in infarct size (12.79 ± 5.97 vs 6.84 ± 1.54% in the controls; ^p^ < 0.05).

Pre-treatment with Mg^2+^ did not prevent or enhance the ISO-induced infarction compared with ISO-treated rats (infarct size: 11.67 ± 6.63%; *p* > 0.05). Treatment with Mg^2+^ alone did not cause injury to the myocardium compared with the controls (infarct size: 6.94 ± 2.1%; *p* > 0.05).

## Effects of ISO and Mg^2+^ on body and organ weights

To examine the systemic and organ-specific effects of the various treatments, the body weight, heart weight and the weights of the other organs were quantified (Table 1). ISO caused a significant increase in the heart weight:body weight ratio compared with the controls (*p* < 0.001). Pre-treatment with Mg^2+^ did not prevent the ISO-induced increase in heart weight:body weight ratio. Compared with the control, ISO also caused a loss in body weight (*p* < 0.05), and pre-treatment with Mg^2+^ did not rectify this weight loss.

**Table 1:** Patient characteristics and demographics

	*Treatment groups*			
*Weight parameter*	*Control (n = 8)*	*ISO (n = 9)*	*ISO + Mg^2+^ (n = 10)*	*Mg^2+^ (n = 8)*
% BW lost	0.54 ± 0.16	–3.65 ± 0.61*	–3.77 ± 1.31*	–0.74 ± 0.79
Heart:BW (mg/g)	3.33 ± 0.06	4.82 ± 0.06***	4.74 ± 0.13***	3.34 ± 0.05
Liver:BW (mg/g)	50.68 ± 1.11	47.34 ± 2.34	44.08 ± 1.32	50.54 ± 1.87
Lung:BW (mg/g)	3.62 ± 0.20	3.21 ± 0.10	3.35 ± 0.10	3.46 ± 0.20
Kidney:BW (mg/g)	6.86 ± 0.10	6.58 ± 0.10	6.38 ± 0.10**	6.57 ± 0.10
Adrenal:BW (mg/g)	0.20 ± 0.01	0.24 ± 0.01	0.23 ± 0.01	0.22 ± 0.01

Body weight: BW. The percentage (%) BW lost was calculated from the differences between the BW measured on the day of treatment and 24 hours later. The organ:BW ratios (mg/g) were based on the BW measured 24 hours post-treatment at organ extraction. Values are mean ± SEM; *p < 0.05; **p < 0.01 and ***p < 0.001 (treatment vs control).

Compared with ISO‑treated rats, Mg^2+^ co-treatment also did not affect the weights or the gross appearances of the liver, lungs, kidneys or adrenal glands. However, co-treatment with ISO and Mg^2+^ significantly decreased the kidney weight:body weight ratio compared to the controls (*p* < 0.01). When Mg^2+^ was administered alone, it did not significantly affect the body weight or the weights of any of the organs compared with the control rats.

## Effects of Mg^2+^ on ISO-induced ECG changes

**Fig. 2.**
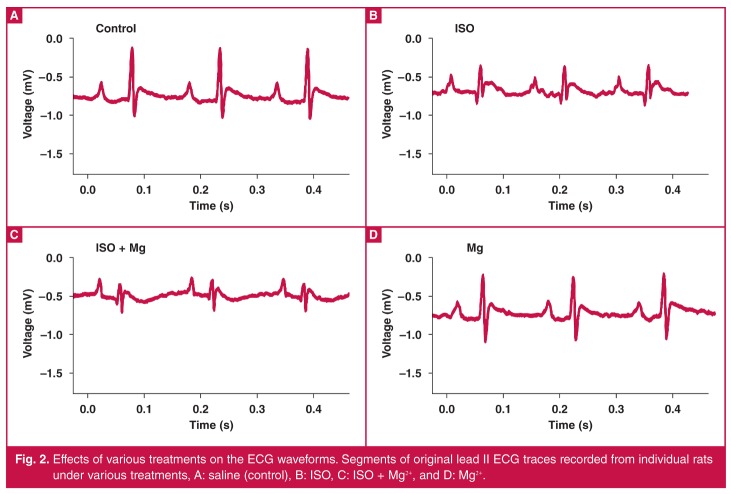
Effects of various treatments on the ECG waveforms. Segments of original lead II ECG traces recorded from individual rats under various treatments, A: saline (control), B: ISO, C: ISO + Mg^2+^, and D: Mg^2+^.

Representative traces of lead II ECG waveforms recorded from individual rats are shown in Fig. 2. Qualitatively, ISO produced a low-voltage ECG recording, with qualitatively large Q waves compared with the controls (Fig. 2A, B). The changes in ECG characteristics produced by various treatments are summarised in Table 2. ISO did not significantly alter the heart rate, the P-wave amplitude and duration, the S-wave amplitude, or the ST-segment height. The drug also had no effects on the PR interval, QRS duration, QT interval or QTc interval.

**Table 2:** Summary data on the effects of chemical treatments on ECG parameters

	Treatment groups			
*ECG parameter*	*Control (n = 8)*	*ISO (n = 9)*	*ISO + Mg^2+^ (n = 10)*	*Mg^2+^ (n = 8)*
Heart rate (bpm)	406.9 ± 9.5	416.6 ± 14.2	418.4 ± 7.2	405.8 ± 15.4
P amplitude (mV)	0.184 ± 0.010	0.165 ± 0.009	0.167 ± 0.014	0.162 ± 0.013
Q amplitude (mV)	–0.024 ± 0.008	–0.111 ± 0.020**	–0.107 ± 0.021*	–0.025 ± 0.008
R amplitude (mV)	0.590 ± 0.056	0.193 ± 0.030***	0.216 ± 0.031***	0.619 ± 0.044
S amplitude (mV)	–0.300 ± 0.073	–0.129 ± 0.060	–0.050 ± 0.022*	–0.248 ± 0.054
T amplitude (mV)	0.123 ± 0.010	0.087 ± 0.009*	0.023 ± 0.018***	0.134 ± 0.008
ST height (mV)	0.054 ± 0.032	0.081 ± 0.008	0.061 ± 0.007	0.109 ± 0.014
P duration (s)	0.184 ± 0.010	0.164 ± 0.009	0.166 ± 0.015	0.161 ± 0.013
PR interval (s)	0.046 ± 0.003	0.050 ± 0.003	0.046 ± 0.002	0.050 ± 0.002
QRS interval (s)	0.014 ± 0.001	0.014 ± 0.001	0.013 ± 0.001	0.015 ± 0.001
QT interval (s)	0.061 ± 0.003	0.046 ± 0.005	0.056 ± 0.007	0.054 ± 0.002
QTc (s)	0.157 ± 0.009	0.123 ± 0.016	0.148 ± 0.017	0.140 ± 0.004
T_peak_-T_end_ (s)	0.040 ± 0.003	0.025 ± 0.003*	0.024 ± 0.003*	0.030 ± 0.001

ECG parameters were sampled from lead II recordings. QTc was calculated using Bazett’s formula. Values are mean ± SEM; *p < 0.05; **p < 0.01 and ***p < 0.001 (treatment vs control).

ISO, however, decreased the R-wave amplitude compared with the controls (*p* < 0.001), an effect consistent with the low-voltage ECG waveform illustrated in Fig. 2B. ISO also produced prominent Q waves compared with the controls (*p* < 0.01), suggesting the presence of an evolving infarct.^38^ The drug also altered ventricular repolarisation by significantly decreasing both the T-wave amplitude and the T_peak_–T_end_ interval compared with the controls (*p* < 0.05 in each case).

Pre-treatment with Mg^2+^ did not affect the ISO-induced changes in ECG voltage or the heart rate, P-wave amplitude and duration, PR interval, QRS duration, QT interval, QTc interval, or the ST-segment height. However, Mg^2+^ decreased the S-wave amplitude (*p* < 0.05), and further decreased the T-wave amplitude (*p* < 0.001) in ISO‑treated hearts compared with the controls, but without causing further changes to the T_peak_–T_end_. interval. Although not statistically significant, as illustrated in Fig. 2C, Mg^2+^ also tended to reduce the size of the Q waves induced by ISO. Mg^2+^ alone did not affect the ECG characteristics compared with those in control rats.

## Effects on haemodynamic parameters

**Fig. 3.**
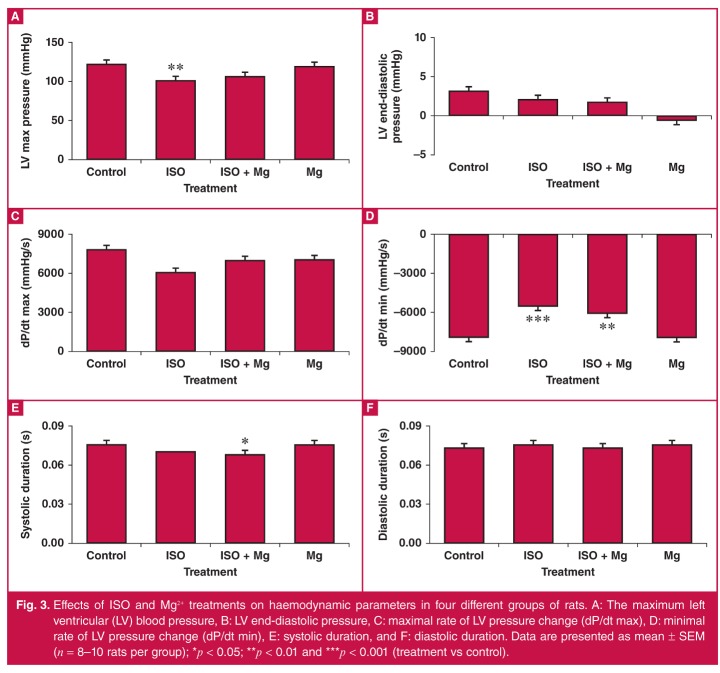
Effects of ISO and Mg^2+^ treatments on haemodynamic parameters in four different groups of rats. A: The maximum left ventricular (LV) blood pressure, B: LV end-diastolic pressure, C: maximal rate of LV pressure change (dP/dt max), D: minimal rate of LV pressure change (dP/dt min), E: systolic duration, and F: diastolic duration. Data are presented as mean ± SEM (*n* = 8–10 rats per group); *p < 0.05; **p < 0.01 and ***p < 0.001 (treatment vs control).

The effects of chemical treatments on haemodynamic parameters are shown in Fig. 3. ISO significantly decreased the left ventricular (LV) maximum blood pressure (101.7 ± 2.2 vs123.4 ± 4.5 mmHg in the controls; *p* < 0.01), but not the LV end-diastolic blood pressure. Mg^2+^ pre-treatment did not reverse or worsen the ISO-induced decrease in the LV maximum blood pressure (107.4 ± 6.4 mmHg; compared with the controls, *p* = 0.34; compared with ISO-treated, *p* = 0.98), or affect the LV end-diastolic blood pressure. ISO significantly decreased the minimal rate of LV pressure change (dP/dt min; –5479 ± 203 vs –7921 ± 435 mmHg/s in controls; *p* < 0.001), but not the maximal rate of LV pressure change (dP/dt max). Mg^2+^ pre-treatment did not reverse the ISO effects on dP/dt min or change the dP/dt max. Mg^2+^ pre-treatment did not affect diastolic duration, but decreased the systolic duration in ISO-treated rats. Mg^2+^ alone did not alter LV blood pressures, LV maximal/minimal dP/dt, or systolic/diastolic duration.

## Effects of ISO and Mg^2+^ on markers of lipid peroxidation

**Fig. 4.**
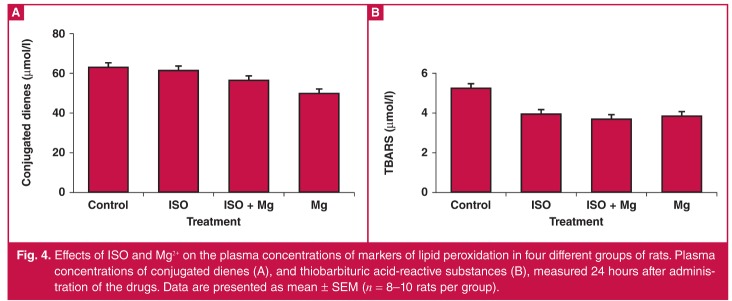
Effects of ISO and Mg^2+^ on the plasma concentrations of markers of lipid peroxidation in four different groups of rats. Plasma concentrations of conjugated dienes (A), and thiobarbituric acid-reactive substances (B), measured 24 hours after administration of the drugs. Data are presented as mean ± SEM (*n* = 8–10 rats per group).

Plasma CD and TBARS were measured 24 hours post-treatment to evaluate the effects of ISO and Mg^2+^ on oxidative stress. Fig. 4 shows that ISO did not alter CD and TBARS plasma concentrations significantly, suggesting that infarction occurred early, in which case the measured concentrations of CD and TBARS may not have reflected the concentrations of these markers at the time of infarction. In addition, Mg^2+^ pre-treatment prior to ISO or treatment with Mg^2+^. alone did not alter the concentrations of these markers.

## Discussion

Despite the advances in modern medical therapy, the mortality rate due to MI remains high. In this study, we used a catecholamine-induced MI model and found that Mg^2+^ prophylaxis did not alter the infarct size, as quantified by TTC staining. Mg^2+^ also had no effect on the ISO-induced ventricular hypotension or disruption of electrophysiological signals. Therefore, while Mg^2+^ did not worsen MI, the preconditions for its therapeutic indications remain unclear.

Several animal models have been developed to mimic human MI *in vivo*, but the lack of reliability, reproducibility or survival remains a problem. Surgical methods such as ligation and cauterisation of coronary arteries produce well-demarcated infarcts compared to the more global infarcts due to ISO (Fig. 1). However, surgical techniques are invasive and associated with post-operative mortality rates as high as 40–50% within 24 hours, and the infarct sizes also vary.^39^

Pharmacological methods such as ISO-induced MI are non-invasive and the drug doses can be adjusted to minimise mortality. However, the methods produce diffuse global infarcts of variable sizes. The ISO-induced MI disease model mimics cardiovascular stress disorders that not only produce infarction, but in which intracellular Mg*2+* deficiency may play a role.^31^,^32^ Nevertheless, in our study, Mg*2+* pre-treatment did not alter infarct size, suggesting a lack of Mg*2+* cardioprotection, as also reported in other studies,^13^,^14^ and at the same time, contradicting the results of some previous studies.^7^-^11^

The moderate dose of ISO used in our study was optimal to induce infarcts and to minimise mortality in our rats. However, the relatively smaller infarcts induced (~15% of the whole ventricular tissue), compared to coronary ligation models (~50% of the localised region at risk),^7^,^8^,^11^ may have made it more difficult to identify any mild effects of Mg^2+^, especially with the presence of baseline infarcts that are attributable to tissue handling.

The protective effects of Mg^2+^ may depend on the dose, bioavailability, and the timing of administration as well as on the type of experimental protocol used. In the experimental studies showing Mg^2+^ protection, Mg^2+^ was given during reperfusion,^7^-^11^ which is a different protocol from the one used in our study. In some studies, Mg^2+^ was protective only when administered early during reperfusion.^8^,^9^ By contrast, Mg^2+^ may have preconditioned the myocardium through the activation of ATP-dependent K^+^ channels,^22^ and also protected it in a cellular model of ischaemia alone without reperfusion.^25^

In our study, serum Mg^2+^ was not measured, making it uncertain whether adequate prophylaxis may have been achieved at the onset of MI. However, a similar dose of Mg^2+^ used in other studies in rats achieved neuroprotection,^35^,^40^ and lower doses used in guinea pigs provided cardioprotection.^41^ In studies where repeated doses of Mg^2+^ were used, it was re-administered only after four hours,^40^ a longer period than when ISO was given in our study. Furthermore, in guinea pigs, Mg^2+^ cardioprotection occurred even if the insult was given at a time when Mg^2+^ levels in the plasma and heart tissue were no longer significantly elevated,^41^ indicating that the downstream cellular effects from the adequate initial exposure to Mg^2+^ may outlast the real elevation of Mg^2+^ in the tissue or plasma.

The low-voltage ECG induced by ISO administration was possibly due to the infarct-related loss of tissue, whereas the presence of pathological Q waves are indicative of an evolving MI.^38^ We however did not observe an elevation of the ST segment, in contrast to what would be expected in acute infarction, and what has been reported by others.^42^ The ST segment in rats is difficult to assess because the end of the QRS complex merges with the T wave (Fig. 2), thereby overshadowing the isoelectric portion.^43^ Therefore, the decreases in the S- and T-wave amplitudes by ISO and Mg^2+^ in our study may in fact reflect ST-segment modulation. Overall, Mg^2+^ pre-treatment did not reverse the electrical changes, in keeping with the unaltered infarct size.

In this study, although Mg^2+^ did not worsen the haemodynamic parameters, it did not reverse the ISO-induced left ventricular hypotension or the decrease in ventricular dP/dt min. The physiological significance of the decrease in the systolic duration in ISO and Mg^2+^ co-treated rats is unclear, but given that the heart rate was unchanged, it is unlikely to be of major impact. By contrast, in an ISO-induced cardiac dysfunction model in dogs, Mg^2+^ preserved ventricular activity and the effect was proposed to be due to Mg^2+^-mediated reduction in cardiac afterload.^19^ In human heart MI, unrelated to ISO, the preservation of ventricular function by Mg^2+^ has been attributed to the direct action of increased extracellular Mg2+ due to MI-induced efflux of Mg^2+^.^44^

It has previously been reported that the action of ISO on the myocardium involves the production of reactive oxygen species,^27^,^28^ and that Mg^2+^ acts as an antioxidant and reduces the infarct size by protecting against free radicals.^16^ In our study, neither ISO nor Mg^2+^ administration resulted in any significant changes in the concentration of oxidative stress markers (CD and TBARS) in the circulation after 24 hours.

After ischaemic damage to myocardial tissue, the blood will reflect the appearance of specific cardiac markers as well as relatively non-specific markers such as those attributable to lipid peroxidation. The latter products, for example CD, lipid hydroperoxides and TBARS depend not only on oxidative stress, but also on the nature of the lipid substrate. Lipid peroxidation products generally change in concert, and therefore using two of the three commonly used markers should reveal the trend. These changes have been reported up to eight days in ISO-induced MI,^45^ and therefore the 24-hour interval in our study could be expected to be appropriate.

Although infarcts were clearly demonstrated in our study, they did not affect the lipid peroxidation markers, suggesting that the infarct size was either too small to produce detectable markers in the plasma, or the lipid substrate was not very susceptible to oxidative stress. By contrast, in coronary artery ligation-induced infarction in dogs, the markers of lipid peroxidation reached peak concentrations a few hours after the ligation.^46^ In rats injected with ISO at a higher dose (110 mg/kg ip, once daily for two days), Anandan *et al*.^47^ found significant increases in the concentration of TBARS in homogenised heart tissue.

With ISO being a broad-acting catecholamine, it is not unexpected for it to produce systemic effects such as the loss of body weight seen in this study. The ISO-related weight loss was previously attributed to the stress of myocardial necrosis or to the catabolic state of altered protein metabolism.^48^ The increase in heart weight:body weight ratio with ISO may indicate the onset of cardiac hypertrophy^49^ or oedema. The increase in heart weight was unlikely to be an artifact related to loss of body weight because the relative weights of the other organs were unaltered by ISO treatment alone. The physiological relevance of the decreased kidney weight in ISO and Mg^2+^ co-treated rats is unclear.

## Conclusion

Our results suggest a lack of reduction in infarct size by single-dose Mg^2+^, despite the presumed optimal pre-treatment. In future studies, it would be important to evaluate serum, heart tissue or urine Mg^2+^ levels to better understand the temporal effects, and to use repeated doses of Mg^2+^ for more sustained prophylaxis. Although Mg^2+^ did not have adverse cardiovascular effects, the role and indications for Mg^2+^ therapy in MI still require further clarification.
